# Supplementary material for: A CXCR4 targeting peptide delivered by silica nanoparticles eliminates migrating cancer stem cells in pancreatic ductal adenocarcinoma
Source: Sci Rep. 2026 Apr 16;16:12588. doi: 10.1038/s41598-026-48584-2 (PMC13087252; doi:10.1038/s41598-026-48584-2)

Figure 3I

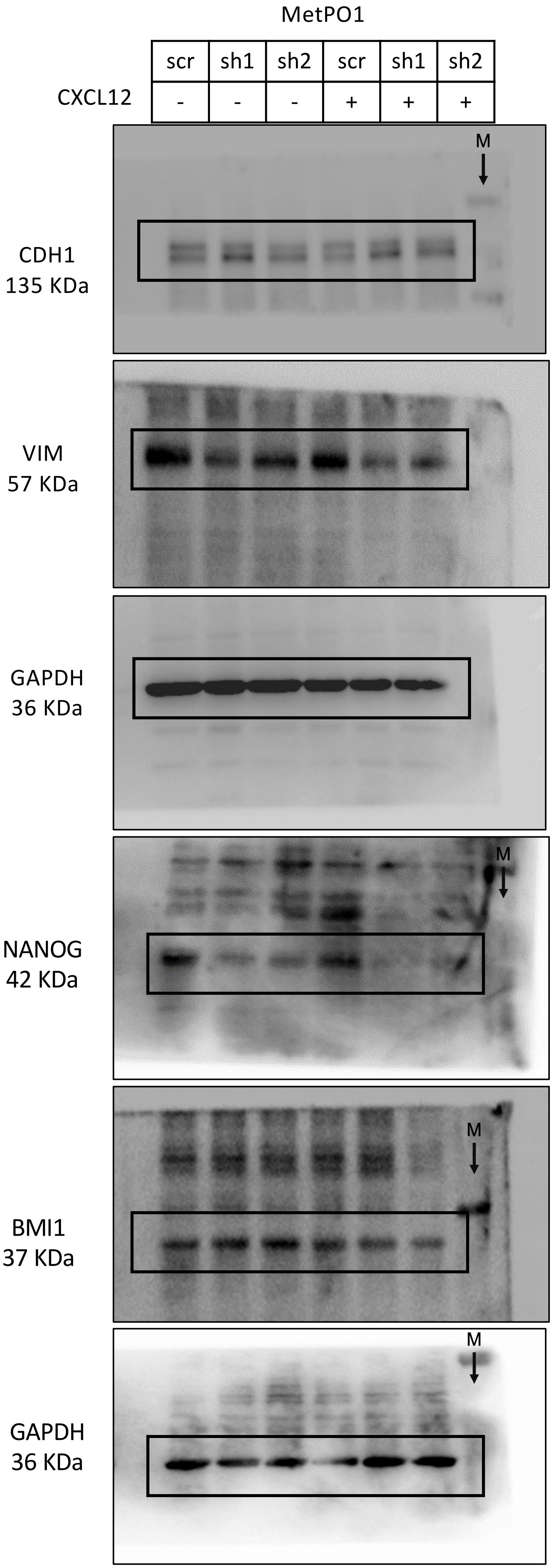
MetPO1
scr
sh1
sh2
scr
sh1
sh2
CXCL12
-
-
-
+
+
+
M
CDH1
135 KDa
VIM
57 KDa
GAPDH
36 KDa
NANOG
42 KDa
M
BMI1
37 KDa
M
GAPDH
36 KDa
M

Figure 3J

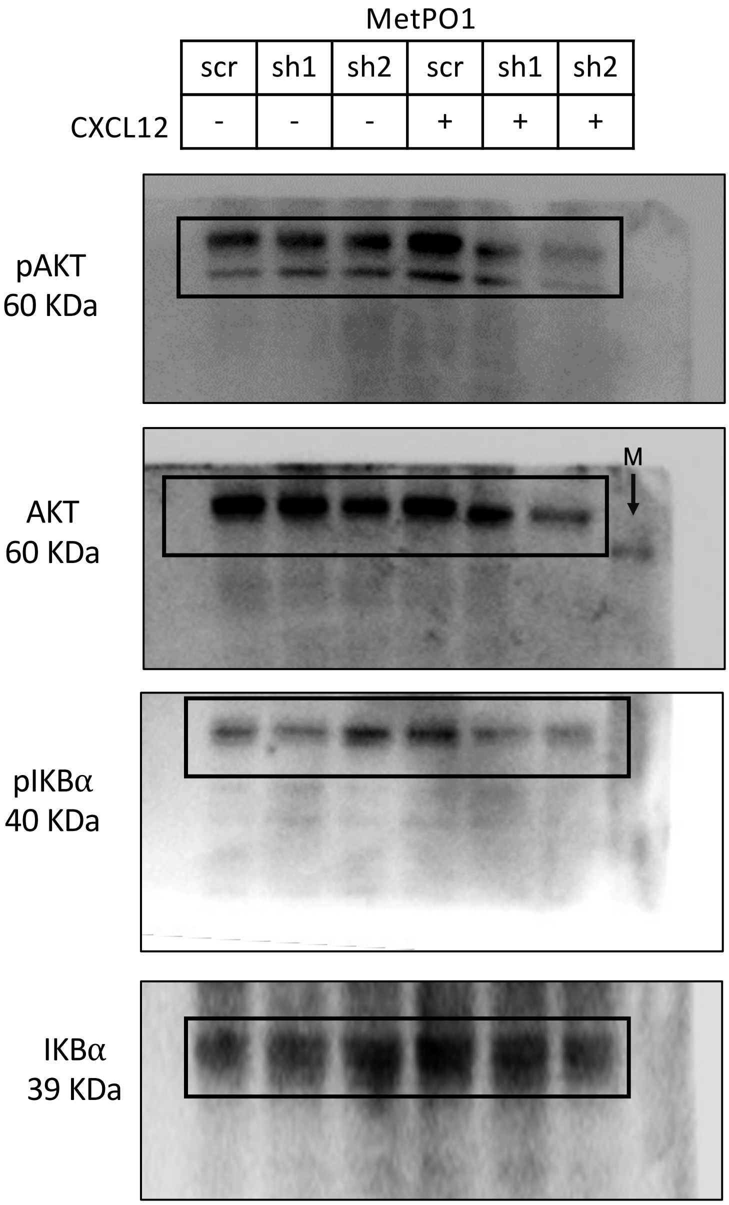
MetPO1
scr
sh1
sh2
scr
sh1
sh2
CXCL12
-
-
-
+
+
+
pAKT
60 KDa
AKT
60 KDa
M
pIKBα
40 KDa
IKBα
39 KDa

Figure 4B

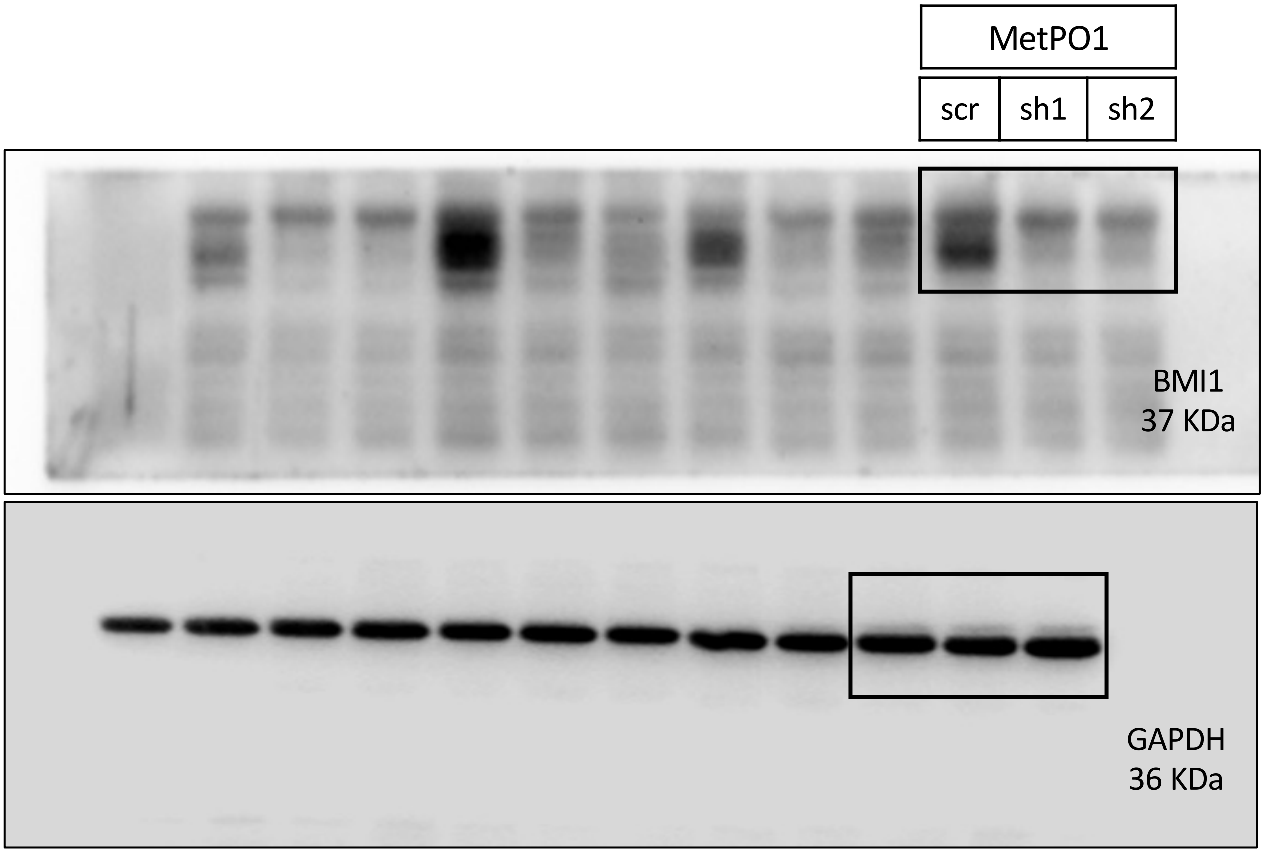

Figure 5H

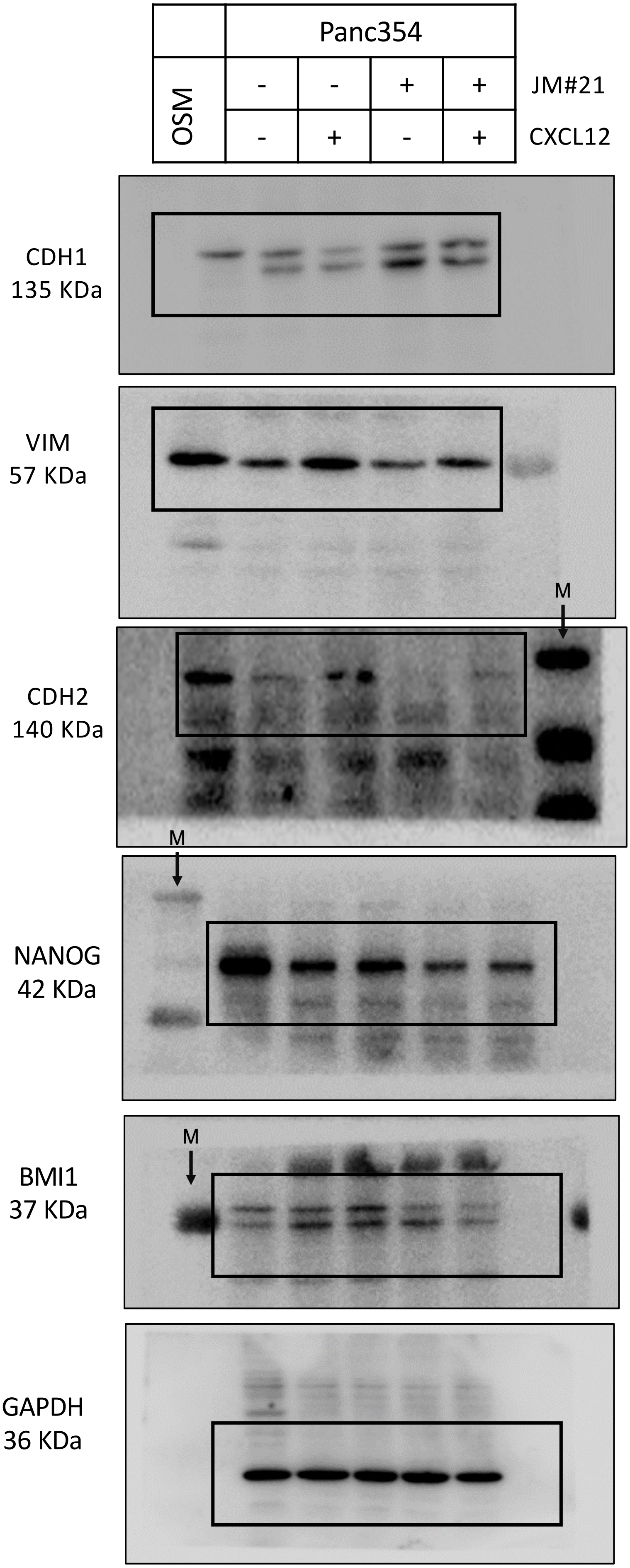

Panc354
OSM
- - + + JM#21
- + - + CXCL12
CDH1
135 KDa
VIM
57 KDa
M
CDH2
140 KDa
M
NANOG
42 KDa
M
BMI1
37 KDa
GAPDH
36 KDa

Supp. Figure 1H

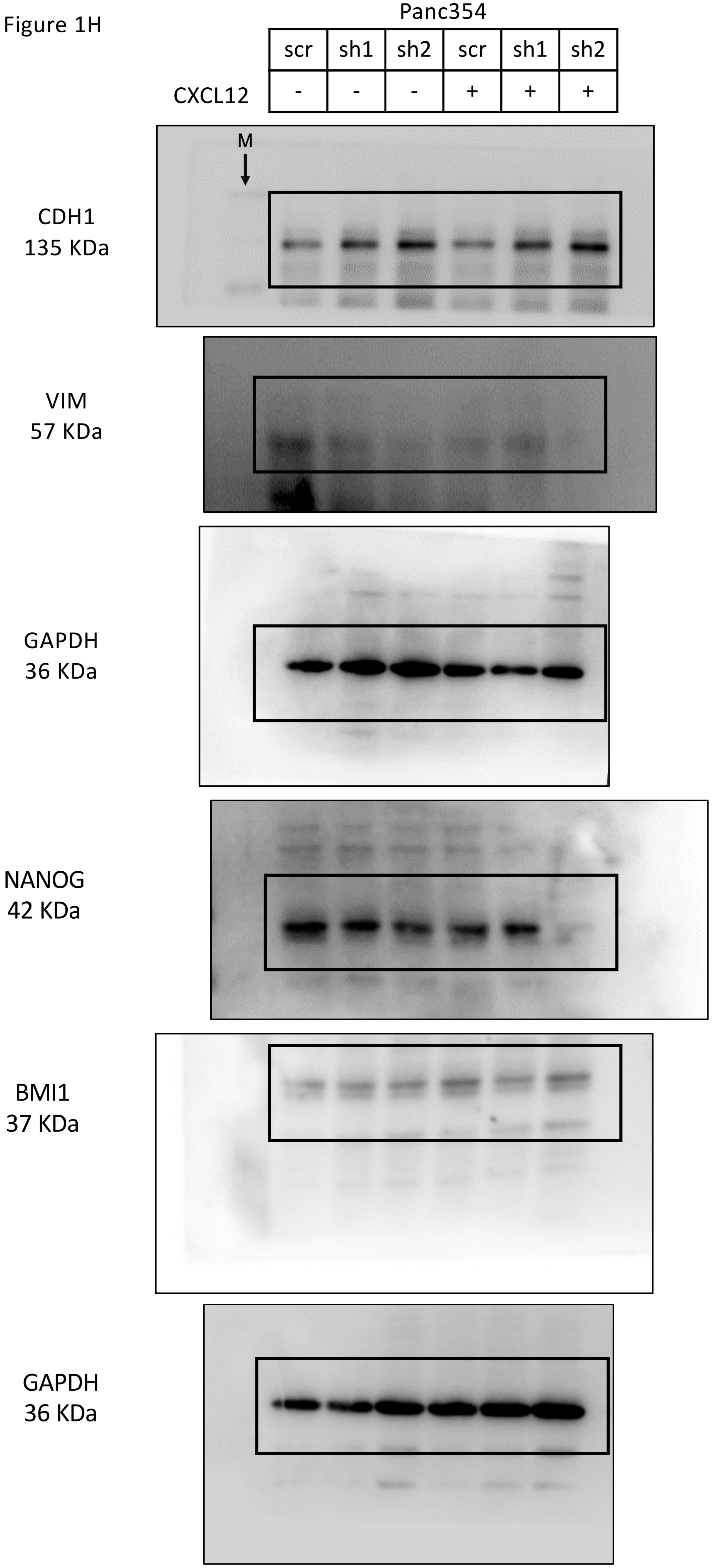

Supp. Figure 1B

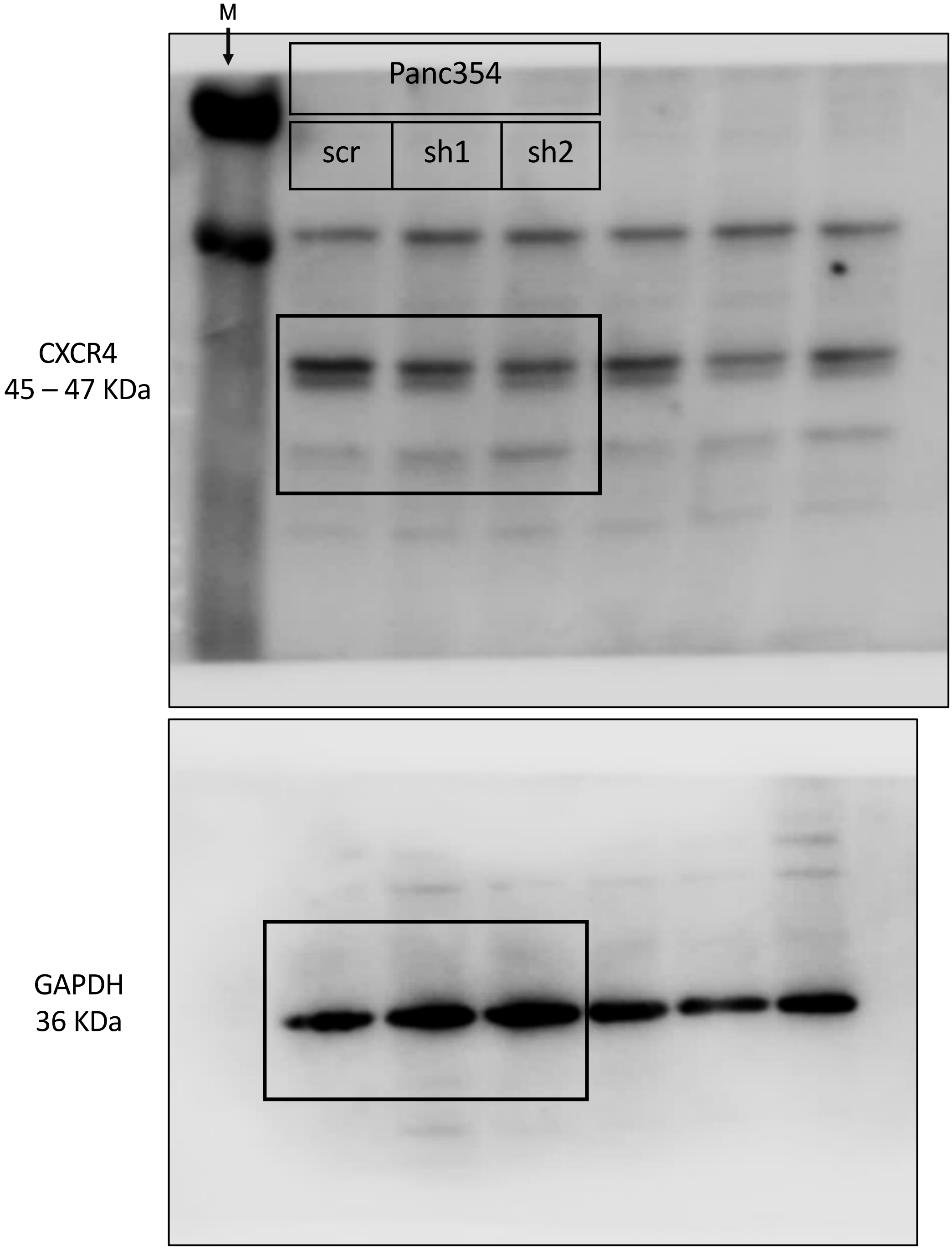

Supp. Figure 1G

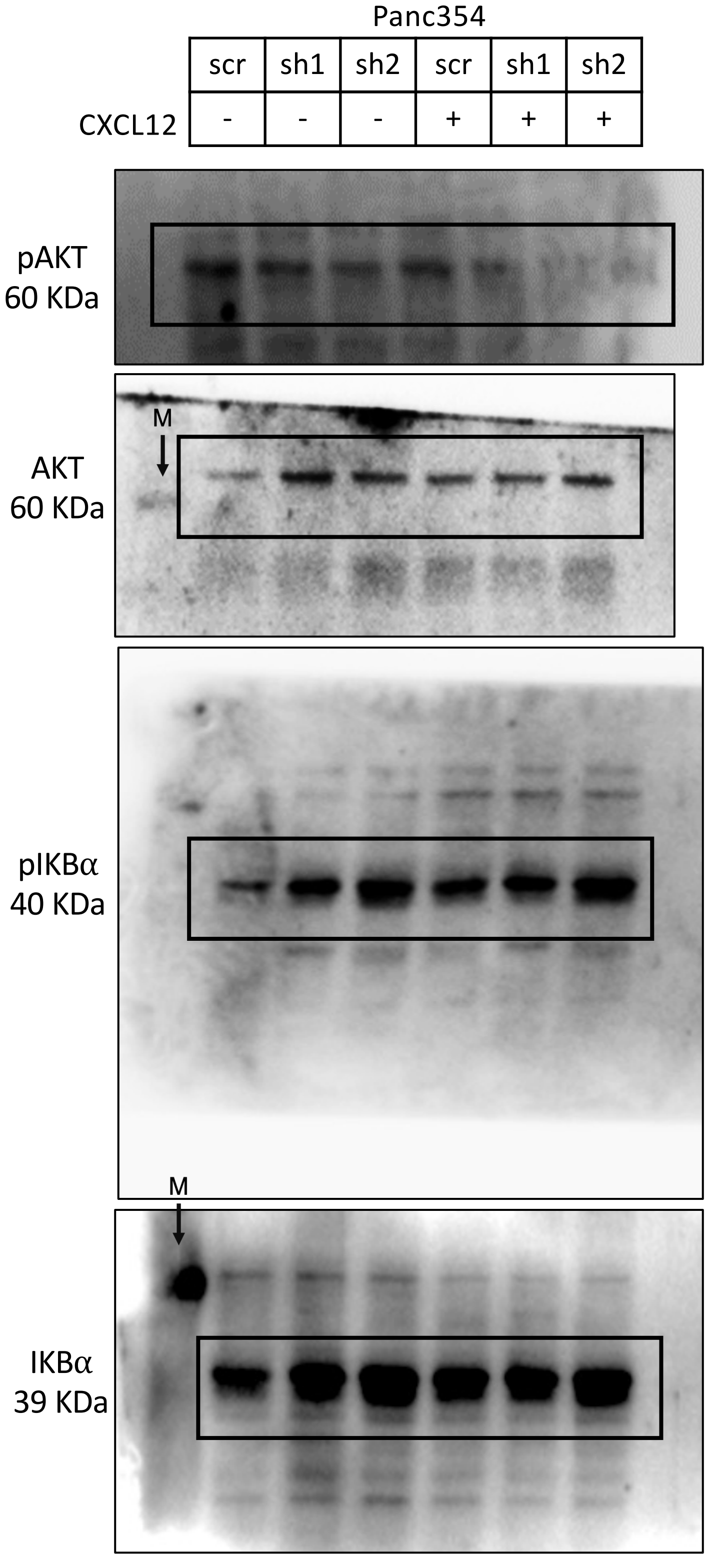

Supp. Figure 2A

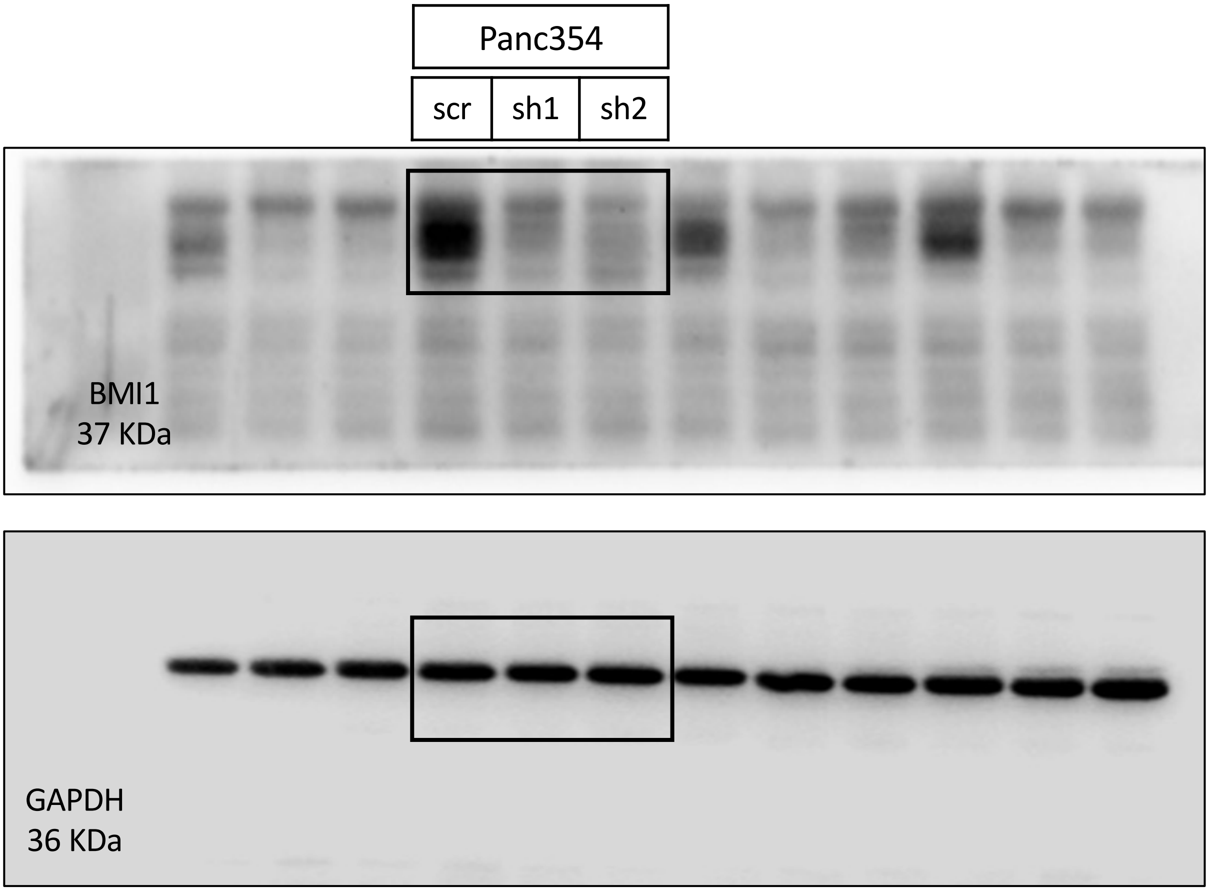

Supp. Figure 2D

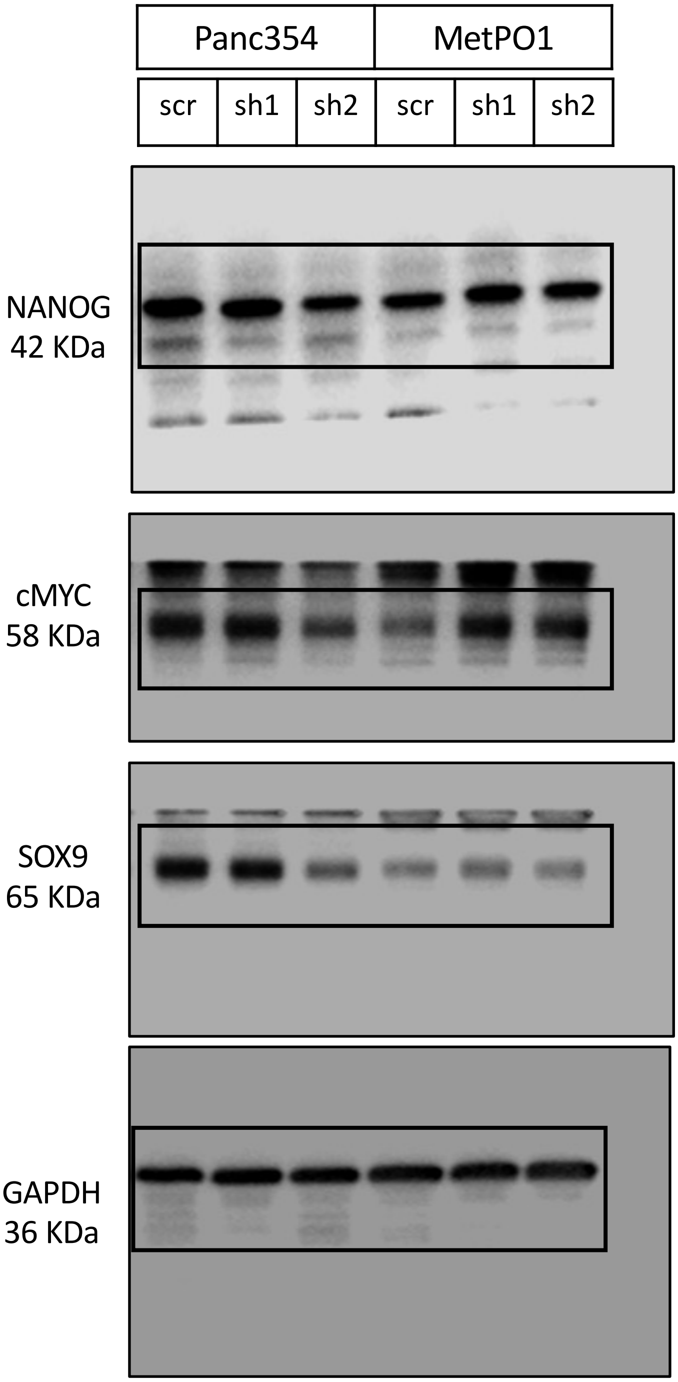

Supp. Figure 2G

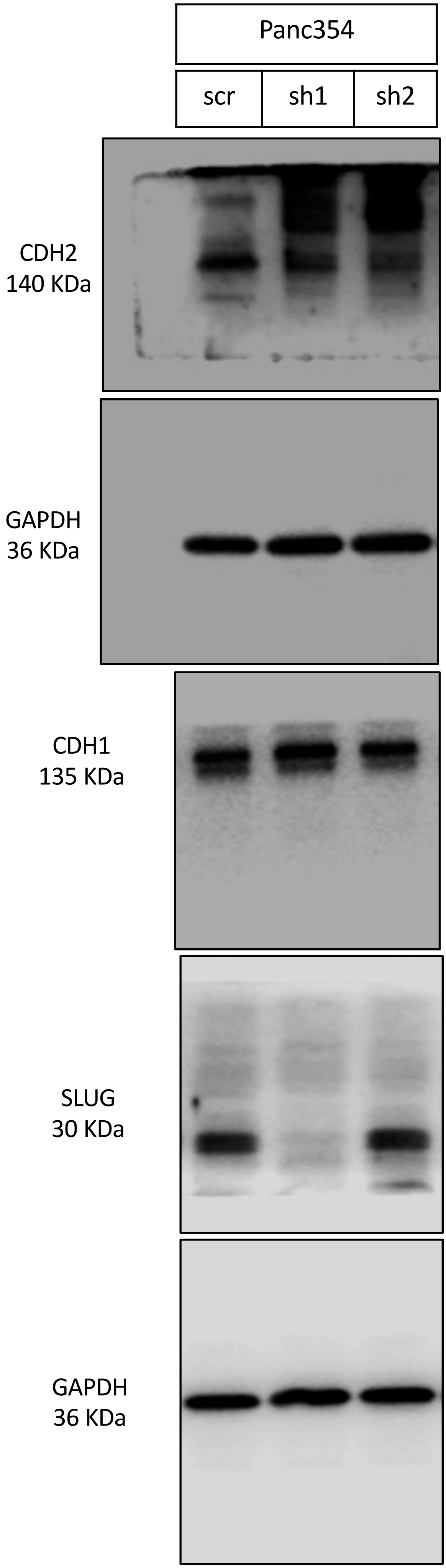

Supp. Figure 3D

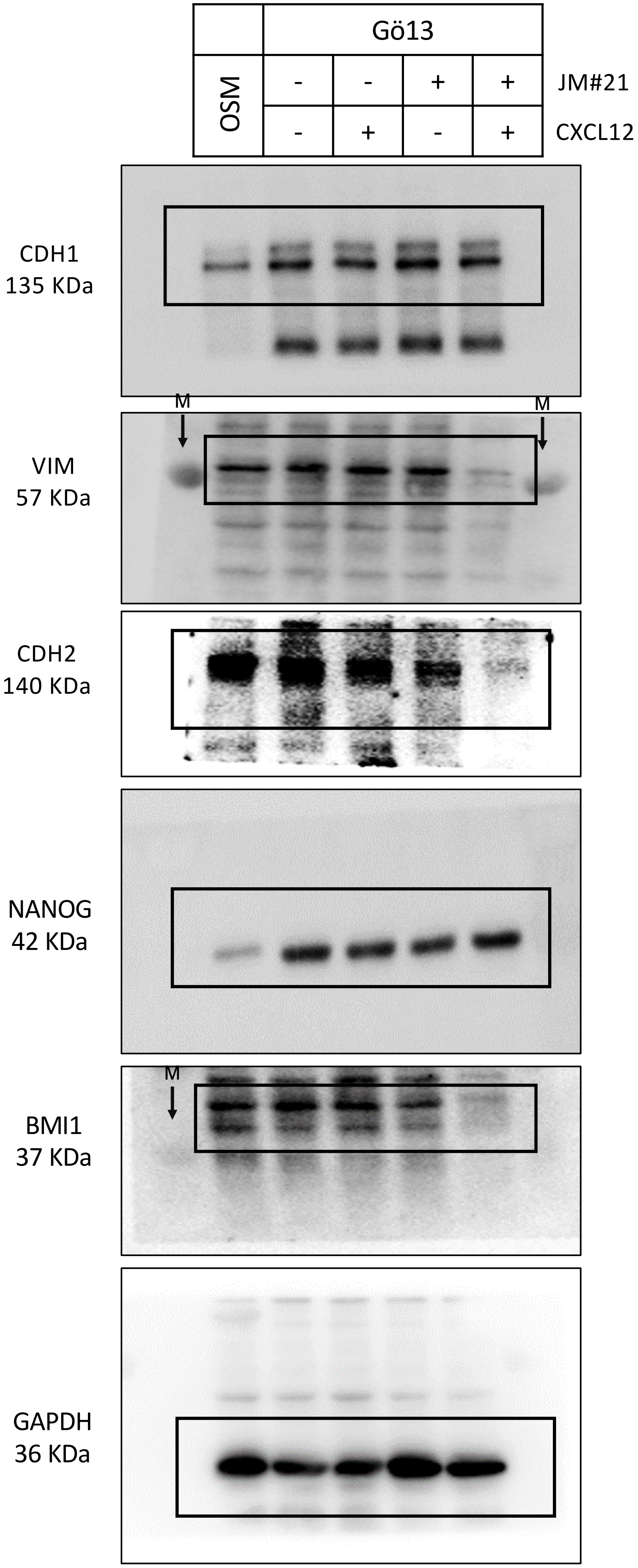

Supp. Figure 3D

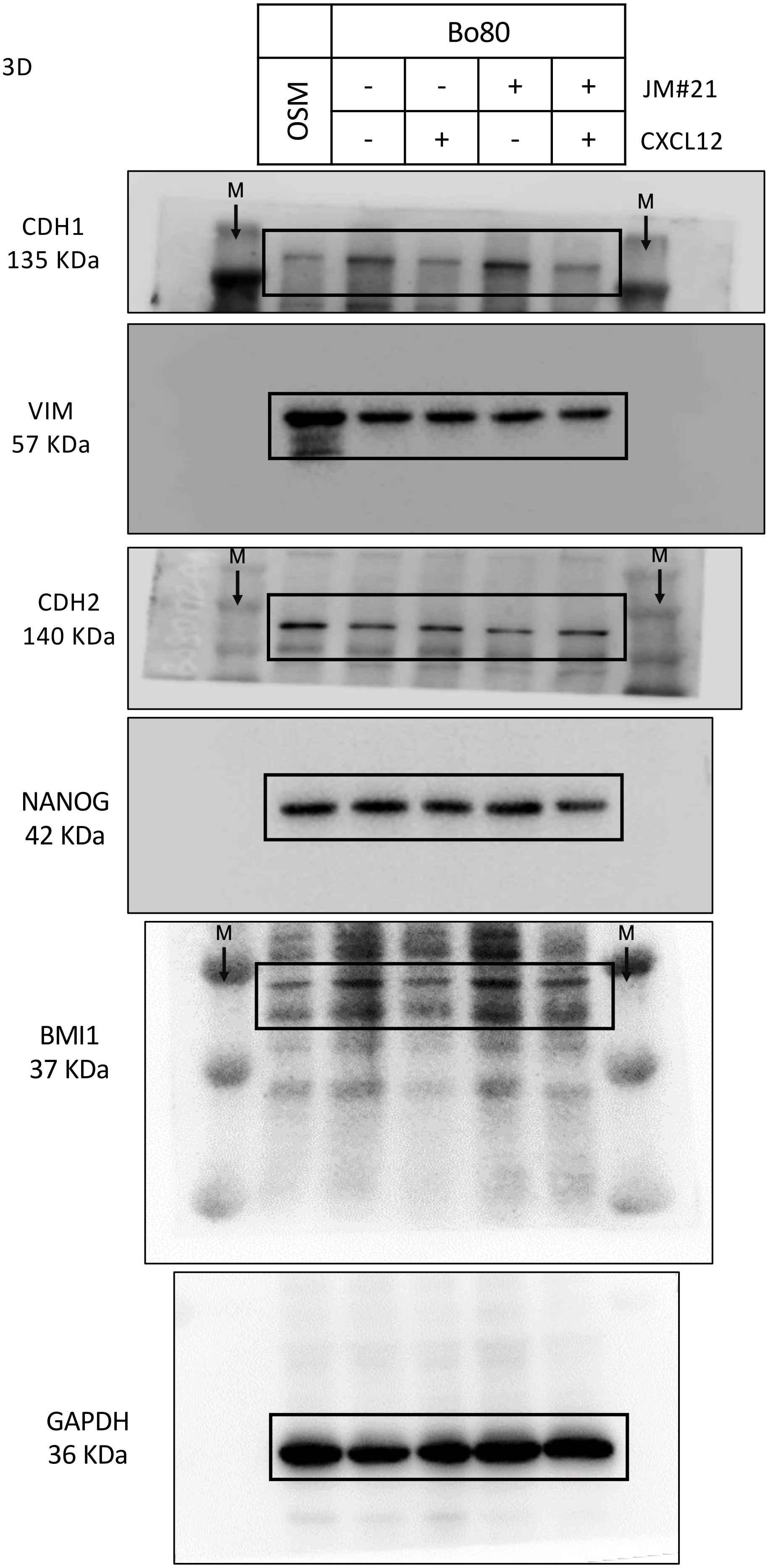

Supplement: Supplementary file 1 — Supplementary Material 1. [file 41598_2026_48584_MOESM1_ESM.pdf]
