# Supplementary material for: A CXCR4 targeting peptide delivered by silica nanoparticles eliminates migrating cancer stem cells in pancreatic ductal adenocarcinoma
Source: Sci Rep. 2026 Apr 16;16:12588. doi: 10.1038/s41598-026-48584-2 (PMC13087252; doi:10.1038/s41598-026-48584-2)
Supplement: Supplementary file 2 — Supplementary Material 2. [file 41598_2026_48584_MOESM2_ESM.docx]

**
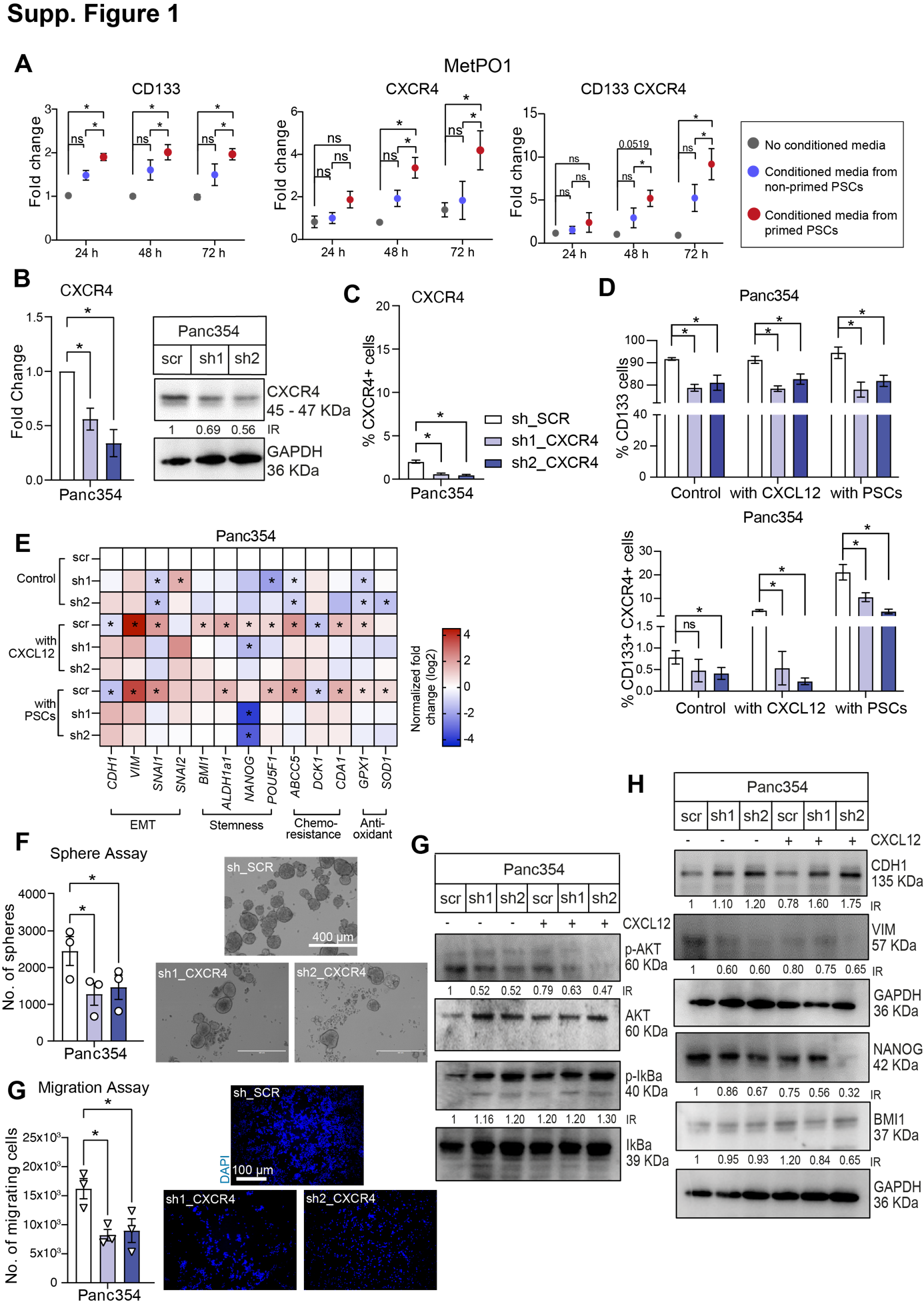
**

**Supplementary Figure 1.**

(A) Flow cytometry analysis performed on MetPO1 when exposed to no conditioned media (grey), conditioned media from non - primed PSCs (blue) or conditioned media from primed PSCs (red) for CD133+ cells, CXCR4+ cells and CD133+ CXCR4+ cells represented as fold change against no conditioned media. (B) *CXCR4* gene expression analysis and western blot analysis in Panc354. GAPDH was used as a loading control. (C) Flow cytometry analysis of CXCR4 surface expression. (D) Flow cytometry analysis of CD133+ CSCs and CD133+ CXCR4+ miCSCs in Panc354 (sh_*SCR*, sh1_*CXCR4* and sh2_*CXCR4*) treated with (or without) CXCL12 or co-cultured with PSCs. (E) Targeted gene expression analysis for indicated genes in Panc354 (sh_*SCR*, sh1_*CXCR4* and sh2_*CXCR4*) treated with (or without) CXCL12 or co-culture with PSCs. (F) Sphere formation assay and representative pictures after CXCL12 treatment in Panc354. (G) Migration assay towards CXCL12 and representative pictures in Panc354. (H) Western blot analysis of labelled protein markers for Panc354 cell line and treatment conditions. GAPDH was used as a loading control. Intensity ratios (IR) calculated against control lane using ImageJ. Cropped blot for clarity. Error bars represent the standard deviation. n=3 for all experiments unless otherwise depicted in the datasets. *p < 0.05, ns = not significant.


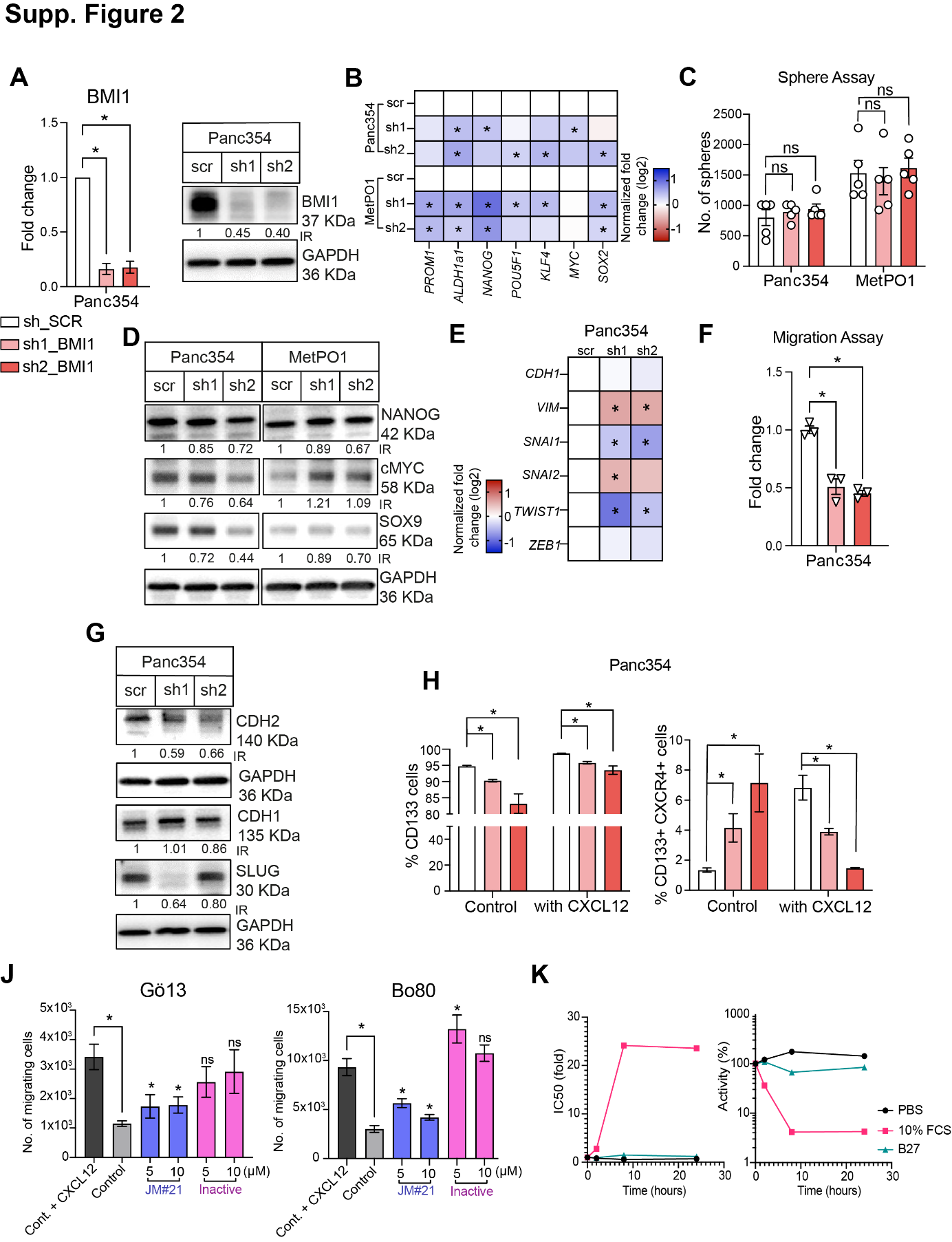


**Supplementary Figure 2.**

(A) *BMI1* gene expression analysis and western blot analysis in Panc354. GAPDH was used as a loading control. Intensity rations (IR) calculated against control lane using ImageJ. Cropped blot for clarity (B) Gene expression analysis for indicated cell lines with genes involved in stemness using qRT-PCR. (C) Sphere formation assays for depicted cell lines. (D) Western blot analysis of NANOG, c-MYC and SOX9 for indicated cell lines. GAPDH was used as a loading control. Intensity ratios (IR) calculated against control lane using ImageJ. Cropped blot for clarity. (E) Gene expression analysis for Panc354 cell line with genes involved in EMT using qRT-PCR. (F) Migration assays towards serum containing media. (G) Western blot analysis of CADHERIN-1, CADHERIN-2 and SLUG for Panc354 cell line. GAPDH was used as a loading control. Intensity ratios (IR) calculated against control lane using ImageJ. Cropped blot for clarity. (H) Flow cytometry analysis of CD133+ cells and CD133+ CXCR4+ cells in Panc354 (sh_*SCR*, sh1_*BMI1* and sh2_*BMI1*) treated with (or without) CXCL12. (J) Migration assays towards CXCL12 for indicated cell lines using JM#21 and the inactive peptide at depicted concentrations. (K) Peptide stability assays for JM#21 in PBS, 10% FCS and B27 to detect activity up to 24 hours. n=3 for all experiments unless otherwise depicted in the datasets. *p < 0.05, ns = not significant.

**
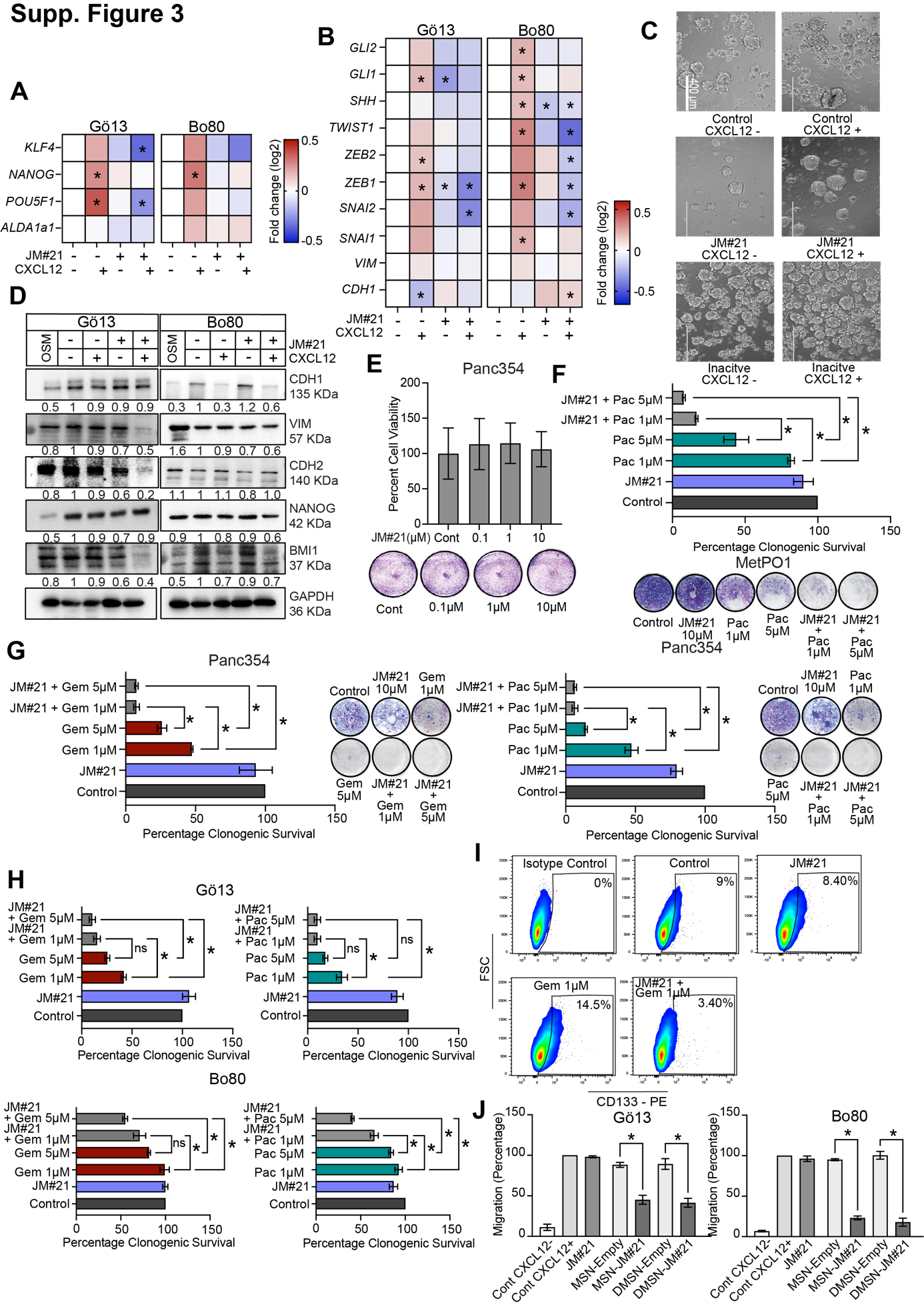
**

**Supplementary Figure 3.**

(A) Gene expression analysis for indicated cell lines with genes involved in stemness. (B) Gene expression analysis for indicated cell lines with genes involved in EMT and SHH pathway. (C) Representative micrographs of sphere cultures after 7 days in Panc354 cells after the indicated treatments. JM#21 and inactive peptide pre-treatment was applied for 30 minutes, respectively. (D) Western blot analysis of CADHERIN-1, VIMENTIN, CADHERIN-2, NANOG and BMI1 for indicated cell lines. GAPDH was used as a loading control. Intensity ratios (IR) calculated against control lane using ImageJ. Cropped blot for clarity. (E) Quantification of cell viability and representative pictures for clonogenic assays after 48 hours of JM#21 treatment for CXCL12 pretreated Panc354 cell line. (F) Quantification of cell viability and representative pictures for clonogenic assays after treatment with JM#21 (10*μ*M), gemcitabine (Gem) or paclitaxel (Pac) for indicated concentrations as depicted in experimental design in MetPO1, (G) in Panc354 and (H) Gö13 and Bo80 cell lines. (I) Representative cytometry plots for Panc354 cells. (J) Migration assays towards CXCL12 in FBS-containing medium with the indicated compounds tested in Gö13 and Bo80 cell lines. n=3 for all experiments unless otherwise depicted in the datasets. *p < 0.05, ns = not significant.
